# Treatment of Decompensated Alcoholic Liver Disease

**DOI:** 10.4061/2011/219238

**Published:** 2011-07-12

**Authors:** John Menachery, Ajay Duseja

**Affiliations:** Department of Hepatology, Post Graduate Institute of Medical Education and Research, Chandigarh 160012, India

## Abstract

Alcoholic liver disease (ALD) is a spectrum ranging from simple hepatic steatosis to alcoholic hepatitis and cirrhosis. Patients with severe alcoholic hepatitis can have clinical presentation almost similar to those with decompensated cirrhosis. Scoring with models like Maddrey discriminant function, a model for end-stage liver disease, Glasgow alcoholic hepatitis score, and Lille model are helpful in prognosticating patients with ALD. One of the first therapeutic goals in ALD is to induce alcohol withdrawal with psychotherapy or drugs. Most studies have shown that nutritional therapy improves liver function and histology in patients with ALD. The rationale for using glucocorticoids is to block cytotoxic and inflammatory pathways in patients with severe alcoholic hepatitis. Pentoxifylline, a tumor necrosis factor alpha (TNF**α**) suppressor, and infliximab, an anti-TNF**α** mouse/human chimeric antibody, has been extensively studied in patients with alcoholic hepatitis. Liver transplantation remains the definitive therapy for decompensated cirrhosis/alcoholic hepatitis despite the issues of recidivism, poor compliance with postoperative care, and being a self-inflicted disease.

## 1. Introduction

Alcohol is a major risk factor for chronic disease burden all over the world. Alcohol abusers and patients with alcoholic liver disease (ALD) usually suffer negative consequences from drinking such as significant financial burden, unemployment, loss of family, accidental injury, or death [1]. Alcoholism is a physical dependence that includes impaired control, craving, development of tolerance, and development of withdrawal symptoms on abstinence.

ALD is a spectrum that ranges from fatty liver to alcoholic steatohepatitis (ASH) and eventually cirrhosis. Simple hepatic steatosis is the commonest histological finding and occurs in 90% of heavy drinkers but is rapidly reversible with abstinence. Alcoholic hepatitis or ASH occurs in up to 35% of heavy drinkers and is usually a precursor of cirrhosis [2].

Epidemiological data suggest that a threshold of 80 g of daily alcohol in a male and 20–40 g in a female for an average of 10 to 12 years is necessary for causing significant alcohol-induced liver injury [3, 4]. However, only a minority of individuals who consume alcohol in excess develop significant ALD. Synergistic factors such as chronic hepatitis C, obesity, and genetic factors may accelerate the development of ALD even at lower doses of alcohol consumption.

ASH is a clinic-pathological syndrome that denotes hepatocellular necrosis and inflammation. The clinical spectrum can range from being asymptomatic to developing overt liver failure. There may be low-grade fever, jaundice, leukocytosis, and mild elevation of transaminases. Histological features of ASH include the presence of parenchymal necrosis, Mallory bodies, and a perivenular neutrophilic infiltrate. Other features that are commonly present include bridging necrosis, fatty changes, bile duct proliferation, cholestasis, and perivenular fibrosis. Liver biopsy as a means of prognostication in alcoholic hepatitis has mostly been replaced with less invasive scoring systems. Patients with severe alcoholic hepatitis can have clinical presentation almost similar to those with decompensated cirrhosis, and it may become difficult to establish if such patients have associated cirrhosis or not. But histologically, the majority of patients with severe alcoholic hepatitis have either significant fibrosis or cirrhosis liver. And alcoholic hepatitis with underlying cirrhosis is one of the most important causes of acute on chronic liver failure (ACLF) [5].

## 2. Prognostic Models in Patients with Alcoholic Liver Disease

### 2.1. Discriminant Function

The Maddrey discriminant function (DF) score remains the most commonly used predictive model and was developed to facilitate the assessment of response in a clinical trial of corticosteroids in patients with alcoholic hepatitis [6]. Modified DF is calculated as = 4.6 × (prolongation of prothrombin time in seconds) + Serum bilirubin (mg/dL). A modified DF score >32 in the presence of hepatic encephalopathy predicts >50% mortality within 28 days in patients with alcoholic hepatitis [7, 8]. However, fatal outcomes have also been known to occur in patients with modified DF score <32, and this low specificity has suggested a need for alternative scoring systems.

### 2.2. Model for End-Stage Liver Disease (MELD)

MELD score was initially developed to predict survival in patients with cirrhosis undergoing transjugular intrahepatic portosystemic shunting (TIPSS). However, it has been found to predict mortality in ASH, and a MELD score ≥21 (within 24 hours of presentation) is a good predictor of 90-day mortality in these patients [9]. MELD and modified DF scores (calculated within 24 hours of presentation) are equivalent in predicting 30- and 90-day mortality in patients with alcoholic hepatitis [10]. 

### 2.3. Glasgow Alcoholic Hepatitis Score (GAHS)

GAHS was developed in an effort to overcome the low specificity of the Maddrey DF and lack of an optimal predictive cutoff point for the MELD score. GAHS is a composite scoring system based on age, serum bilirubin, blood urea nitrogen, PT, and the peripheral leucocyte count. GAHS ≥9 is a predictor of mortality and is more accurate than DF in predicting both 28- and 84-day mortality but is equivalent to MELD in predicting 28-day mortality [11].

### 2.4. Lille Model

The Lille model incorporates age, renal insufficiency, albumin, PT, bilirubin, and the evolution of bilirubin on day 7 to predict 6-month mortality in patients with severe alcoholic hepatitis who have received corticosteroid therapy [12].

A recent study showed that among the various prognostic scores for acute alcoholic hepatitis (Lille, Glasgow, and Maddrey scores) and cirrhosis (MELD, MELD-Na, and Child-Turcotte-Pugh) in ALD patients treated with corticosteroids, Lille score ≥0.45 and GAHS ≥9 were the most accurate models for the prediction of mortality [13]. Although the components may be different in each of these scores, they help the physicians to identify a subset of patients with higher mortality and requiring aggressive management.

## 3. Treatment of Alcoholic Liver Disease

### 3.1. General Management

One of the first therapeutic goals of patient management in patients with ALD is to induce alcohol withdrawal. The administration of fluid, calories, vitamins, and minerals is usually required. However, overhydration should be avoided, as this can worsen ascites and can precipitate variceal bleed. Vitamin K is usually administered to patients who have a prolonged prothrombin time, even though this regimen is typically ineffective because coagulopathy reflects severity of underlying liver disease. Correction of the coagulopathy with fresh frozen plasma is not recommended in the absence of active hemorrhage, because this treatment might increase the risk of variceal hemorrhage in a patient with portal hypertension. Admission to a critical care unit should be considered for unstable patients, and airway protection should be assured in a patient with hepatic encephalopathy.

### 3.2. Abstinence

Abstinence is the cornerstone of therapy in the management of ALD. Ideally, this includes rehabilitation with a multidisciplinary approach. If abstinence is achieved, clinical and histological improvement occurs, even if the patient is already cirrhotic [14–17]. Both psychological and pharmacological approaches can be used to treat alcohol dependence. Psychological interventions involve strategies to educate and inform patients about the nature of their problem and provide them with advice on how to change their behavior. Psychosocial treatments such as cognitive behavioral therapy and motivational enhancement therapy have been shown to reduce alcohol intake in alcohol-dependent patients [18].

As an addition to psychological therapies, many patients might benefit from pharmacological therapy. Both acamprosate and naltrexone have been demonstrated to reduce the number of drinking days and increase abstinence rates in randomized controlled trials [19]. Acamprosate, unlike naltrexone, is well tolerated except in patients with Child C cirrhosis and its benefit seems to persist for at least 1 year after treatment withdrawal. Disulfiram, an inhibitor of acetaldehyde dehydrogenase, has been used for many years in the management of alcohol-dependent patients, although with conflicting results. 

### 3.3. Nutrition

Patients with ALD are malnourished for a number of reasons, including malabsorption, the induction of a catabolic state, and the replacement of calories with alcohol. Protein-caloric malnutrition has also been demonstrated to correlate with short-term and long-term mortality in alcoholics. Hence, malnutrition should be actively sought in such patients, and replacement should be commenced accordingly. The efficacy of nutritional therapy in ASH has been evaluated in numerous clinical trials. Although various results have been reported, most studies have shown that nutritional therapy improves liver function and histology. Enteral feeding is preferred to parenteral nutrition. Although protein ingestion is a theoretical risk factor for the development of hepatic encephalopathy, protein feeding is well tolerated, and protein should not be routinely restricted in patients with ASH.

Two randomized controlled trials have looked at the effects of nutritional therapy. The first study compared enteral tube feeding of an energy-dense formula (>2,000 kcal daily) with an isocaloric standard oral diet in 35 randomly allocated, severely malnourished, cirrhotic patients. In-hospital mortality was significantly lower in the enteral group (12% versus 47%) [20]. The second study which compared enteral feeding with steroids in 71 patients with acute, severe ASH found that there was no difference in mortality between the groups during the 28-day treatment period. However, the mortality rate was lower in the enterally fed group in the year following treatment [21]. In summary, nutritional supplementation could have a role in the improvement of medium-term to long-term survival in patients with severe ASH. The American College of Gastroenterology guidelines recommend 1.2 to 1.5 g/kg of protein and 35 to 40 kcal/kg of body weight for patients with ALD [22].

### 3.4. Glucocorticoids

Glucocorticoids are the most intensely studied and yet most hotly debated treatment for acute alcoholic hepatitis. The rationale for glucocorticoid use is to block cytotoxic and inflammatory pathways in alcoholic hepatitis. Glucocorticoids have been shown to decrease proinflammatory cytokines and intercellular cell adhesion molecule 1 expression and inhibit neutrophil activation and have demonstrated short-term histological improvement in patients with alcoholic hepatitis.

Results from trials of glucocorticoids for ALD are variable and depend on the nature of the trial and the group of patients recruited as the study population. Even among glucocorticoids trials with beneficial results, enrolled subjects were heterogeneous with variable definitions of randomization and blinding and without homogeneous inclusion or exclusion criteria. Different types of steroids for different durations and different criteria were used for treatment. Steroid use in alcoholic hepatitis raises the risk of infection in an already immunocompromised host. Some trials have demonstrated higher mortality in the glucocorticoid group compared to the placebo group [23–26]. Associated with this higher mortality, a greater incidence of fungal infections among patients receiving glucocorticoids has been reported by some authors [25]. A meta-analysis on this subject, published in 1990, demonstrated a protective effect of glucocorticoids in high quality trials. This was especially so in studies that excluded patients with gastrointestinal bleeding but included those with hepatic encephalopathy [27]. But another meta-analysis by Christensen and Gluud found no benefit once they attempted to control for confounders [28]. A subsequent reanalysis of the same 3 randomized, controlled trials in Christiansen and Gluud's meta-analysis, which pooled raw data from more than 200 patients with modified DF ≥32, found a 28-day survival benefit of glucocorticoids (85%) versus placebo (65%). In patients with modified DF ≥32, treatment with glucocorticoids improved short-term (28-day) survival, with mortality decreasing from 35% in controls to 15% with steroids. Conversely, patients with modified DF <32 had a >90% survival rate without steroids. The number of patients who needed to be treated to save 1 patient was 5 [29]. Another meta-analysis of 15 trials with 721 randomized patients reported that the evidence in favor of glucocorticoids was based on heterogeneous trials of low quality [30]. Recently using individual patient data from more than 400 patients, Mathurin et al. demonstrated improved survival with corticosteroid treatment. Patients were classified as complete responders (Lille score ≤0.16; ≤35th percentile), partial responders (Lille score 0.16–0.56; 35th–70th percentile), and null responders (Lille ≥0.56; ≥70th percentile). 28-day survival was strongly associated with these groupings (91% versus 79% versus 53%, *P* < 0.0001). Corticosteroids had a significant effect on 28-day survival in complete responders and in partial responders but not in null responders [31]. The long-term benefit of steroids is difficult to assess as the various trials had differing follow-up periods, and unless the patient abstains from alcohol completely, alcoholic hepatitis is likely to recur. The survival benefit of corticosteroid therapy has not been found to persist beyond 1 year.

Despite having 13 randomized controlled trials and 6 meta-analyses of steroids as a treatment for ASH, concerns over their use continue. Although corticosteroids are probably beneficial in patients with severe disease, mortality on treatment remains high, particularly when renal impairment is present, and treatment is contraindicated in a relatively large number of patients with concomitant infection and gastrointestinal bleeding.

### 3.5. Antitumor Necrosis Factor Alpha Treatment

#### 3.5.1. Pentoxifylline

Elevated tumor necrosis factor alpha (TNF**α**) levels have been found to be predictive of poor survival in patients with alcoholic hepatitis. Pentoxifylline is a nonselective phosphor-di-esterase inhibitor and a TNF**α**  suppressor. In 1991, a study of pentoxifylline for severe alcoholic hepatitis (DF ≥ 32) reported a reduction in the development of hepatorenal syndrome and mortality in comparison with placebo [32]. A subsequent study of 101 patients from the same center supported the earlier findings, demonstrating a 40% reduction in mortality in comparison with placebo. The number needed to treat to prevent 1 death was 4.7. However, in this study, there was no demonstrable improvement in routine liver function tests or liver histology and the better survival was predominantly due to decreased incidence of hepatorenal syndrome [33]. 

In another study, 29 patients who did not respond to corticosteroids (identified by an absence of an early decline in bilirubin) were switched to pentoxifylline for 28 days and compared to 58 other matched patients who persisted with corticosteroid therapy. No survival benefit was observed with pentoxifylline at 2 months [34]. Thus, although some data suggest a benefit with pentoxifylline in alcoholic hepatitis, it is unclear whether its benefit extends beyond possibly preventing hepatorenal syndrome.

#### 3.5.2. Inflximab

Infliximab is an anti-TNF**α**  mouse/human chimeric antibody and has been extensively studied in alcoholic hepatitis. Early reports were encouraging, demonstrating improved survival rates, improved Maddrey scores, or improved laboratory parameters. However, the largest randomized, controlled trial to date, which enrolled 36 patients and compared a combination of prednisolone (40 mg/day) and infliximab (10 mg/kg 3 times per week in weeks 0, 2, and 4) to prednisolone and placebo in alcoholic hepatitis was terminated prematurely [35]. More deaths had occurred in the group treated with prednisolone and infliximab. Whether this risk was related to the higher doses of infliximab used, more ill patients being recruited in this trial in comparison with earlier studies, the combined use of prednisolone and infliximab, or the possible unsuitability of infliximab for the treatment of alcoholic hepatitis is still debatable.

#### 3.5.3. Etanercept

A small, uncontrolled study evaluated the use of etanercept in 13 patients with moderate to severe alcoholic hepatitis. Two patients died within 32 days, and 3 developed serious adverse events (infection, gastrointestinal bleeding, and hepatorenal syndrome) mandating withdrawal of the drug [36]. A more recent multicenter, randomized, double-blind, placebo-controlled study of etanercept in 48 patients with severe alcoholic hepatitis (defined as MELD ≥ 15) found no difference in the 1-month mortality rates in the 2 groups on an intention-to-treat analysis [37]. Alarmingly, the 6-month mortality in the etanercept group was significantly higher in comparison with the controls. Rates of serious adverse infectious events were also significantly higher in the etanercept group. These results have considerably dampened the enthusiasm for using anti-TNF**α**  agents in patients with severe ASH.

### 3.6. Antioxidant Cocktails

Antioxidants have been tried in alcoholic hepatitis as oxidative stress is a key factor in its pathogenesis. However, results have failed to show any convincing benefit from their use. Phillips et al., who compared corticosteroids to an antioxidant cocktail (**β**-carotene, vitamins C and E, selenium, methionine, allopurinol, desferrioxamine, and *N*-acetylcysteine), reported inferior survival rates in comparison with corticosteroids at 30 days [38]. Another study stratified patients with severe alcoholic hepatitis by gender and corticosteroid treatment. The active group received a loading dose of *N*-acetylcysteine (150 mg/kg followed by 100 mg/kg/day for 1 week) and daily doses of vitamins A and E, biotin, selenium, zinc, manganese, copper, magnesium, folic acid, and coenzyme Q for 6 months. Antioxidant therapy showed no benefit, either alone or in combination with corticosteroids [39]. A more recent study of 87 patients with severe alcoholic hepatitis (defined as modified Maddrey DF ≥32) randomized patients to receive either corticosteroids with *N*-acetylcysteine infusion for 5 days or corticosteroids alone [40]. Although patients in the *N*-acetylcysteine group had better 1-month survivals, this effect did not persist at 3 and 6 months.

### 3.7. S-Adenosyl Methionine

*S*-Adenosyl methionine (SAMe) is a precursor of glutathione that theoretically might be protective in alcohol-induced liver injury. In a Cochrane database review of 9 randomized trials that combined a heterogeneous sample of 434 patients with ALD, SAMe failed to show a survival benefit [41]. Only 1 trial of 62 patients deemed to have adequate methodology and outcome reporting and good quality suggested benefit (improved survival and delay to liver transplantation) with 2 years of SAMe treatment for Child's class A and B alcoholic cirrhosis [42].

### 3.8. Treatment of Hepatorenal Syndrome Associated with Alcoholic Hepatitis

In patients with severe ASH, the development of renal failure is associated with a survival of less than 10% even with intensive management. The most significant advance in the management of patients with advanced liver disease over the past decade has been the introduction of albumin infusions combined with splanchnic vasoconstrictor agents for patients with hepatorenal syndrome. Although no randomized trials have specifically examined this form of therapy in patients with ASH, the previously reported high mortality in ASH patients with hepatorenal syndrome suggests that albumin infusions combined with splanchnic vasoconstrictor agents would have a significant and beneficial effect on patient survival. The usefulness of pentoxyfylline in this clinical setting has been mentioned earlier.

### 3.9. Liver Transplantation in ALD

Orthotopic liver transplantation (OLT) remains the definitive therapy for decompensated cirrhosis due to ALD despite continued alcohol abstinence. Short-term (1- to 7-year) graft survival and patient survival remain at par with, if not superior to, survival with non-ALD if the patient remains abstinent. Bellamy et al. reported that in 123 patients who were transplanted, patient survival at 1, 5, and 7 years was 84%, 72%, and 64%, respectively. Graft survival was 81%, 66%, and 50%, respectively, over the same period [43]. The 1- and 5-year patient and graft survival rates for all patients with cirrhosis were 86.9%/73.4% and 82.4%/67.4%, respectively [44].

However, OLT for ALD patients continues to fuel controversy, including issues of recidivism, potentially poor compliance with postoperative care, and inherent biases against alcoholics, such as concern about using scarce organs for what is often perceived to be a self-inflicted disease.

DiMartini et al.'s prospective study of alcoholic recipients found that 22% had used some alcohol by the end of the first year post-OLT and 42% had by 5 years, of whom 26% had a binge drinking pattern [45]. Such a wide range of relapse rates may stem from varying definitions of recidivism and methods of eliciting alcohol consumption data. Most studies addressing recidivism in the past 20 years have used the “any use” definition of alcohol relapse. However, a return to drinking does not necessarily mean excessive drinking. Furthermore, Fabrega et al.'s report of patients who had returned to drinking revealed no decreased compliance with other medical care, including immunosuppressant therapy [46]. Pfitzmann et al. stratified relapsers into slips and harmful drinking, revealing significantly worse 5- and 10-year survival rates (69.5% and 20%) among “harmful” drinkers versus abstainers (90.3% and 81.5%) [47]. 

A major focus in determining candidacy for liver transplantation in ALD has been identifying factors to predict posttransplant recidivism. Pretransplant duration of abstinence from alcohol was the first predictive factor analyzed. Other medical and social variables can be associated with high relapse rates, including tobacco consumption, noncompliance to follow-up clinic visits, and mental illness. A 2008 meta-analysis of 50 studies looking at predictors of recidivism found 3 significant, albeit modest, variables: a poor social support system, a family history of alcohol abuse/dependence, and pretransplant abstinence of 6 months or less [48].

Pre-OLT abstinence, especially the 6-month rule, remains contentious. Studies over the years have provided convincing data for and against the 6-month abstinence requirement. Lucey et al. suggested that this 6-month period of abstinence would allow the native liver to recover with medical management and possibly obviate transplantation [49]. However, this minimal period of abstinence is sometimes waived if the patient is deemed too ill to survive beyond 6 months without a liver transplant. One study showed that recovery in decompensated alcoholic cirrhosis by alcohol abstinence can be predicted within 3 months of abstinence by the monitoring of clinical signs via the Child-Pugh scoring system (serum bilirubin, albumin, international normalized ratio, ascites, and hepatic encephalopathy) [50]. This study by Veldt et al. found that although such improvement in liver function can take place within 3 months of abstinence, some abstinent patients die within 6 months; this has led some authors to suggest reducing the period of abstinence from 6 to 3 months. Yet, abstinence less than 12 months was recently identified as a significant risk factor for relapse in a large retrospective study of OLT recipients [50]. 

Finally, an increasing concern of late is the high risk for de novo malignancies in long-term survivors transplanted for ALD. Although posttransplantation lymphoproliferative disorder and nonmelanoma skin cancer remain the most common malignancies after liver transplantation, the incidence of esophageal cancer is significantly increased in patients with alcohol as the etiology of end-stage liver disease. Duvoux et al.'s prospective study showed a significantly higher incidence of malignancies in patients with ALD compared to those with non-ALD etiologies [51]. They also detected squamous cell carcinoma of the oropharynx or esophagus only in recipients transplanted for ALD. Risk factors undoubtedly include the cumulative effects of alcohol and, in most cases, smoking with posttransplant immunosuppression. Thus, regular ear, nose, and throat examinations appear justified in patients transplanted for ALD.
